# Mitochondrial Dysfunction in the Pathogenesis of Rett Syndrome: Implications for Mitochondria-Targeted Therapies

**DOI:** 10.3389/fncel.2017.00058

**Published:** 2017-03-14

**Authors:** Natalya Shulyakova, Ana C. Andreazza, Linda R. Mills, James H. Eubanks

**Affiliations:** ^1^Division of Genetics and Development, Krembil Research Institute, University Health NetworkToronto, ON, Canada; ^2^Department of Physiology, University of TorontoToronto, ON, Canada; ^3^Department of Pharmacology, University of TorontoToronto, ON, Canada; ^4^Institute of Medical Sciences, University of TorontoToronto, ON, Canada; ^5^Department of Surgery (Neurosurgery), University of TorontoToronto, ON, Canada

**Keywords:** Rett syndrome, MECP2, mitochondrial dysfunction, oxidative stress

## Abstract

First described over 50 years ago, Rett syndrome (RTT) is a neurodevelopmental disorder caused primarily by mutations of the X-linked *MECP2* gene. RTT affects predominantly females, and has a prevalence of roughly 1 in every 10,000 female births. Prior to the discovery that mutations of *MECP2* are the leading cause of RTT, there were suggestions that RTT could be a mitochondrial disease. In fact, several reports documented altered mitochondrial structure, and deficiencies in mitochondrial enzyme activity in different cells or tissues derived from RTT patients. With the identification of *MECP2* as the causal gene, interest largely shifted toward defining the normal function of MeCP2 in the brain, and how its absence affects the neurodevelopment and neurophysiology. Recently, though, interest in studying mitochondrial function in RTT has been reignited, at least in part due to observations suggesting systemic oxidative stress does play a contributing role in RTT pathogenesis. Here we review data relating to mitochondrial alterations at the structural and functional levels in RTT patients and model systems, and present a hypothesis for how the absence of MeCP2 could lead to altered mitochondrial function and elevated levels of cellular oxidative stress. Finally, we discuss the prospects for treating RTT using interventions that target specific aspects of mitochondrial dysfunction and/or oxidative stress.

## Introduction

Mutations of the gene encoding methyl-CpG-binding-domain-containing-protein 2 (*MECP2*) are the underlying cause for the vast majority of people affected with typical Rett syndrome (RTT; OMIN #312750) (Amir et al., 1999), with mutations of the genes encoding cyclin-dependent kinase-like 5 (*CDKL5*) and forkhead box g1 (*FOXG1*) also causal for other less common RTT cases (Weaving et al., 2004; Ariani et al., 2008). MeCP2 is a pleotropic factor, which depending on context, can repress or stimulate gene expression (Yasui et al., 2007; Chahrour et al., 2008) by regulating both local and higher-order chromatin organization (Jones et al., 1998; Georgel et al., 2003; Skene et al., 2010). Through these mechanisms, MeCP2 functions as a key transcriptional control orchestrator that maintains homeostasis in different cells and systems throughout the body in accordance with local demands (Dani et al., 2005; Kron et al., 2012; Li et al., 2013). Since MeCP2 is globally expressed (Reichwald et al., 2000; Shahbazian et al., 2002), mutations that negatively affect MeCP2 function would likely alter transcriptional control in virtually every cell in the body, albeit to differing levels depending on the level of MeCP2 expression in any given cell, and the prevalence of other systems that can partially compensate for its absence. Given that RTT is a neurological condition (Johnston et al., 2001; Neul and Zoghbi, 2004), it is not surprising that the brain expresses the highest levels of MeCP2 in the body (Shahbazian et al., 2002), and the selective removal of MeCP2 from neurons is sufficient to induce a RTT-like phenotype in mice (Chen et al., 2001; Guy et al., 2001). However, the importance of MeCP2 function in glial cells is also recognized, as its selective ablation from oligodendrocytes induces behavioral impairments in mice (Nguyen et al., 2013), and the reactivation of Mecp2 in only astrocytes of MeCP2-null mice improves aspects of their RTT-like behavior (Lioy et al., 2011). Precisely how this pathology arises remains unclear, but dysregulation of the three-dimensional chromatin organization and proper coordinated gene expression are likely initiating points in the pathogenic cascade. As nuclear genes encode the vast majority of mitochondrial proteins (Gregersen et al., 2012), the possibility that a MeCP2 deficiency could facilitate alterations in the expression patterns of mitochondrial factors cannot be excluded.

Mitochondria are the primary energy producing organelles, and consequently, defects or changes in mitochondrial gene expression patterns can affect normal energy production capacity. Proteins residing within mitochondria are encoded by both nuclear and mitochondrial genomes (Gregersen et al., 2012), and it is now established that MeCP2 either directly or indirectly regulates the expression of several nuclear genes encoding mitochondrial factors (Kriaucionis et al., 2006; Gibson et al., 2010; Pecorelli et al., 2013). Given the ubiquitous expression pattern of MeCP2, any deficit in MeCP2 function could therefore lead to mitochondrial impairments in any cell type of the body. However, tissues with elevated energy demands, such as nerve and muscle, would likely be most susceptible to damage caused by functionally impaired mitochondria (Wong, 2010). In fact, the “Mitochondrial Diseases” are a specific category of disorders that arise from mutations in mitochondrial DNA (mtDNA)- or nuclear DNA (nDNA)-encoded mitochondrial proteins, which often disproportionately affect brain function compared to other organs or systems. Despite being caused by different mutated genes, many of these diseases share major clinical features. These include, but are not limited to, early symptomatic onset, developmental delay, motor and mental regression, dystonia, ataxia, muscle weakness (hypotonia), cardiomyopathy, seizures, gastrointestinal reflux, and impaired function of the respiratory system (Schon and Manfredi, 2003). Mitochondrial diseases are also characterized by oxidative damage, a decrease in electron transport chain (ETC) complex activities, and elevated levels of lactate and pyruvate in blood (Chinnery and Schon, 2003; Mattman et al., 2011).

Rett syndrome shares many common features with mitochondrial diseases, although not generally reaching the same degree of overall severity. In addition to overlaps in their respective clinical presentations, these similarities include elevated levels of lactate and pyruvate in blood and cerebrospinal fluid (Matsuishi et al., 1992; Lappalainen and Riikonen, 1994; Haas et al., 1995), altered electron transport chain (ETC) complex function (Kriaucionis et al., 2006; Gold et al., 2014), and evidence for increased oxidative damage (De Felice et al., 2009, 2011a,b, 2012). Such commonality may not be surprising, as alterations in the expression patterns of certain nuclear genes that are themselves causal for specific mitochondrial disorders when mutated have been observed in RTT patients and model systems (see below). In fact, clear indices of abnormal mitochondrial structure and function have been observed in different cell types derived from both symptomatic MeCP2-deficient mice as well as RTT patients (Tables 1–3). In MeCP2-deficient mice, alterations in mitochondrial function are evident at both early and late stages of symptomatic progression, but differences do exist in their pattern of dysfunction. For example, while both pre-symptomatic and symptomatic MeCP2-null mice display elevated markers for oxidative stress (De Felice et al., 2014), the burden is higher in mice at symptomatic stages (De Felice et al., 2014). Similarly, while mitochondrial membrane potential in hippocampal stratum pyramidale neurons is preserved in brain slices from pre-symptomatic neonatal MeCP2-null mice (Großer et al., 2012), it is significantly decreased (depolarized) in the same cells in symptomatic MeCP2-deficient mice (Großer et al., 2012). These data not only illustrate that altered mitochondrial function is one consequence of MeCP2 dysfunction, but also suggest that there is progressivity in dysfunction as symptomatic severity increases. Further, they raise the possibility that impaired mitochondrial function could indeed play a contributing rather than passive role in RTT pathogenic progression. Finally, the decreased ATP levels that have been observed in the symptomatic MeCP2-null mouse brain (Saywell et al., 2006), in the female Mecp2^308^ mouse brain (De Filippis et al., 2015), and in isolated Mecp2^−/y^ microglia (Jin et al., 2015) provides further evidence that the absence of MeCP2 leads to impaired mitochondrial function.

**Table 1:** **Genes encoding oxidative phosphorylation factors that display altered expression in MeCP2-deficient systems**.

**Gene**	**Coding genome**	**Protein function**	**Δ**	**Cell/tissue type**	**Organism**	**References**
*Mt-Nd2*	mtDNA	NADH-dehydrogenase subunit 2 (complex I)	↓	Brain tissue	MeCP2-null mice	Kriaucionis et al., 2006
*NDUFA 1, 2, 8, 9* *NDUFB 2, 4, 6, 9, 10* *NDUFC 1, 2* *NDUFS 4, 5, 6* *NDUFV 2*	nDNA	NADH: ubiquinone oxidoreductase (complex I)	↑	Lympho-monocytes	Human (RTT patients)	Pecorelli et al., 2013
*SDHB*	nDNA	Succinate dehydrogenase complex, subunit B (complex II)	↑	Lympho-monocytes	Human (RTT patients)	Pecorelli et al., 2013
*Uqcrc1*	nDNA	Cytochrome b-c1 complex subunit1 (complex III)	↑	Brain tissue	MeCP2-null mice	Kriaucionis et al., 2006
*UQCRC1*	nDNA	Cytochrome b-c1 complex subunit1 (complex III)	↓	ESC derived MeCP2-null neurons	Human	Li et al., 2013
*UQCRQ* *UQCRFS1* *UQCRH*	nDNA	Subunits of complex III	↑	Lympho-monocytes	Human (RTT patients)	Pecorelli et al., 2013
*CO1*	mtDNA	Cytochrome c oxidase subunit 1 (complex IV)	↓	Frontal and occipital cortex	Human (RTT patients)	Gibson et al., 2010
				MeCP2-null SH-SY5Y	Human	Gibson et al., 2010
				Skeletal muscles	MeCP2-null mice	Gold et al., 2014
*COX14, COX6C, COX7C, 7A2, 8A*	nDNA	Subunits of cytochrome c oxidase (complex IV)	↑	Lympho-monocytes	Human (RTT patients)	Pecorelli et al., 2013
*ATP5A1, ATP5EP2, ATP5J2, ATP5O*	nDNA	Subunits of ATP synthase (complex V)	↑	Lympho-monocytes	Human (RTT patients)	Pecorelli et al., 2013
*ATPIF1*	nDNA	ATPase inhibitory factor 1	↑	Lympho-monocytes	Human (RTT patients)	Pecorelli et al., 2013
*ANT1*	nDNA	Adenine nucleotide transporter	↑	Brain tissue, cerebellum, skeletal muscles	MeCP2-null mice	Forlani et al., 2010
*CYCS*	nDNA	Electron carrier cytochrome c	↑	Lympho-monocytes	Human (RTT patients)	Pecorelli et al., 2013

**Table 2:** **Genes displaying altered expression in MeCP2-deficient systems that encode mitochondrial structural and organization factors**.

**Gene**	**Coding genome**	**Protein function**	**Δ**	**Cell/ tissue type**	**Organism**	**References**
*HMN*	mtDNA	16sRNA	↑	Brain tissue	Human (RTT patients)	Gibson et al., 2010
*TIMM 8B, 9, 10, 13, 17A* *TOMM7 TSPO*	nDNA	Mitochondrial import and localization	↑	Lympho-monocytes	Human (RTT patients)	Pecorelli et al., 2013
*MRPL13, 20, 21, 33, 51, 52* *MRPS25, 30, 33, 36*	nDNA	Mitochondrial ribosomal proteins	↑	Lympho-monocytes	Human (RTT patients)	Pecorelli et al., 2013
				ESC-derived neurons^1^	Human (RTT patients)	Tanaka et al., 2013
*Pgc-1α*	nDNA	Mitochondrial biogenesis	↓	Skeletal muscles	MeCP2-null mice	Gold et al., 2014
*Crls1*	nDNA	Synthesis of cardiolipin	↓	Skeletal muscles	MeCP2-null mice	Gold et al., 2014
*NR3C1*	nDNA	Mitochondrial transcription factor	↑	ESC-derived neurons	Human (RTT patients)	Tanaka et al., 2013

**Table 3:** **Genes displaying altered expression in MeCP2-deficient systems that encode oxidative defense factors**.

**Gene**	**Coding genome**	**Protein function**	**Δ**	**Cell/ tissue type**	**Organism**	**References**
*SOD1*	nDNA	ROS scavenging enzyme	↑	Hippocampus	MeCP2-null mice	Großer et al., 2012
				Lympho-monocytes	Human (RTT patients)	Pecorelli et al., 2013
*CAT*	nDNA	Catalase	↑	Lympho-monocytes	Human (RTT patients)	Pecorelli et al., 2013
*PRDX1*	nDNA	Peroxiredoxin	↑			
*GSTO1*	nDNA	Glutathione S-transferase omega 1	↑			
*MGST2, MGST3*	nDNA	Microsomal glutathione S-transferase	↑			
*Prdh*	nDNA	Proline dehydrogenase	↑	Brain tissue	MeCP2-null mice	Urdinguio et al., 2008
*Ucp3*	nDNA	Uncoupling protein 3	↓	Skeletal muscles	MeCP2-null mice	Gold et al., 2014

## Evidence for increased oxidative stress in Rett syndrome tissues and model systems

Since mitochondria are the major sites of reactive oxygen species (ROS) production and also contribute to ROS detoxification, mitochondrial dysfunction generally results in excessive ROS production and/or diminished ROS detoxification, which collectively heighten levels of oxidative stress. As mentioned previously, elevated oxidative stress has repeatedly been reported in RTT patients. In Sierra et al. (2001) showed a decrease in the activity of superoxide dismutase in erythrocytes from RTT patients, and an increase in plasma malondialdehyde, a marker of lipid peroxidation. Later, De Felice et al. (2009) also demonstrated the presence of oxidative stress markers such as intra erythrocyte and plasma non-protein-bound iron (NPBI; i.e., free iron), esterified F2-isoprostanes (F2-IsoPs, as free, esterified, and total forms), and protein carbonyls in RTT patients indicative of both systemic oxidative stress and lipid peroxidation. In subsequent studies they also reported the presence of oxidative stress markers in different RTT patient group blood samples (De Felice et al., 2011a, 2012; Leoncini et al., 2011), and elevated levels of oxidative stress markers such as intra erythrocyte and plasma NPBI, plasma F2-IsoPs and 4-hydroxynonenal protein adducts (4-HNE PAs) in additional RTT patients (Ciccoli et al., 2012). More recently Signorini et al. (2014) reported significant increases in F4-Neuroprostanes (F4-NeuroPs), F2-IsoPs, NPBI, and 4-HNE PAs, together with a significant decrease in reduced glutathione (GSH)—one of the major non-enzymatic endogenous antioxidants—in skin fibroblasts from RTT patients. These observations not only illustrate evidence for elevated oxidative damage in RTT patients, but also implicate decreased anti-oxidative defense as a mitigating mechanism.

Similar heightened levels of oxidative stress have also been observed in MeCP2-null mouse models. Elevated mitochondrial respiration rates and a reduction in coupling were detected in MeCP2-null male mouse brains (Kriaucionis et al., 2006). Hippocampal slices from MeCP2-null male mice displayed higher oxidized baseline conditions, increased oxidative burden and reduced activity of the ROS scavenger SOD1 (Großer et al., 2012). Oxidative stress markers (F4-NeuroPs, F2-IsoPs, NPBI, and 4-HNE PAs) were significantly elevated in brain samples of MeCP2-null (pre-symptomatic and symptomatic) and *Mecp2*^*308*^ mutated (pre-symptomatic and symptomatic) male and female mice (De Felice et al., 2014). Interestingly, after reactivation of MeCP2 in the brain of previously MeCP2-null mice, the levels of the oxidative stress markers F4-NeuroPs, F2-IsoPs, and NPBI were restored to wild type levels (De Felice et al., 2014). These results illustrate the increased oxidative burden stems directly from the absence of MeCP2, and importantly, that it is not permanent and can be abrogated. Finally, and consistent with RTT patient data, a decrease in the GSH/GSSG ratio has been observed in the brains of MeCP2-null mice (Szczesna et al., 2014), along with a decrease in GSH levels in skeletal muscles of symptomatic MeCP2-null mice (Gold et al., 2014). Collectively, these data provide further indication that the absence of MeCP2 is causal for a significant increase in oxidative stress indicators in the brain of mice.

## Mitochondrial structure is altered in Rett syndrome patients and model systems

Several studies of mitochondria in different tissues of RTT patients and MeCP2-null mouse models have also identified alterations in mitochondrial ultrastructure. Electron microscopic analyses of muscle and skin biopsies revealed the presence of abnormally swollen and dumb-bell shaped mitochondria with vacuolization, abnormally elongated mitochondria with vacuolization and concentric membranous profiles, and oversized mitochondria that had fewer and shorter cristae than normal (Eeg-Olofsson et al., 1988, 1990; Ruch et al., 1989; Wakai et al., 1990; Dotti et al., 1993; Cornford et al., 1994; Cardaioli et al., 1999). Similar swollen and vacuolated mitochondria have also been seen in sural nerve biopsies from RTT patients (Wakai et al., 1990), and in neurons from the frontal cortex, cerebellum and substantia nigra in postmortem patient samples (Cornford et al., 1994). Analysis of mitochondria from MeCP2-null mice revealed commonalities with the findings in RTT patients. Ultrastructural analysis of neurons within brain slices from 3 week old male *Mecp2*^*tm1.1Bird*^ and *Mecp2*^*tm1.1Jaenisch*^ strains of mice found enlarged, elongated mitochondria with enlarged cristae in hippocampal neurons (Belichenko et al., 2009). Similarly, studies of muscles from 8-week old male *Mecp2*^*tm1.1Jaenisch*^ mice reported enlarged and misshapen mitochondria with electron-lucent central matrices and non-parallel, disorganized, cristae (Park et al., 2014). These observations are not absolute, however, as despite observing significant alterations in mitochondrial function in brain samples taken from 12 week old male *Mecp2*^*tm1.1Bird*^, Kriaucionis et al. (2006) reported the absence of gross structural abnormalities in mitochondria purified total brain relative to age-matched wild-type mice. Thus, while morphological alterations are commonly observed, mitochondria with seemingly normal morphologies can still display pronounced functional deficiencies in MeCP2-deficient tissues. These data suggest that the observed morphological changes are late occurring events in RTT symptomatic progression, and may be a consequence of chronically elevated mitochondrial stress.

## The expression levels of genes and proteins related to mitochondrial function are altered in MeCP2-deficient cells

Nuclear genes encode more than 99% of mitochondrial proteome, with the remaining proteins encoded by the mitochondrial genome (Gregersen et al., 2012). Several studies have reported alterations in the expression profiles of different mitochondrial factor-encoding genes in samples from RTT patients, MeCP2-null mouse models, and MeCP2-deficient cell lines. Although the specific factors identified in each individual study were largely non-overlapping, the collective results suggest the normal homeostasis of mitochondria in MeCP2-deficient cells is indeed altered by the absence of MeCP2. Common themes emerging from these studies were: (1) the upregulated expression of several nuclear-encoded components of ETC Complexes I, II, III, and IV (Kriaucionis et al., 2006; Gibson et al., 2010; Pecorelli et al., 2013); (2) an induction in the expression of genes encoding proteins that regulate mitochondrial structure/organization (Pecorelli et al., 2013); (3) the upregulation of different mitochondrial ribosomal factors involved with translation (Pecorelli et al., 2013); (4) an induction of factors directly involved with maintaining mitochondrial functional homeostasis (Forlani et al., 2010; Tanaka et al., 2013); and (5) an induction of factors involved with anti-oxidative defense (Großer et al., 2012; Pecorelli et al., 2013). In addition to upregulated expression changes, there were also notable mitochondrial factors that showed diminished expression in MeCP2-deficient systems. These include cytochrome c oxidase (complex IV) (Gibson et al., 2010; Gold et al., 2014), the transcriptional regulator PGC-1α, mitochondrial uncoupling protein 3, cardiolipin synthase (Gold et al., 2014), and NADH-dehydrogenase subunit 2 (Kriaucionis et al., 2006). The pattern of change for NADH-dehydrogenase subunit 2 was particularly intriguing, as while decreases were evident in MeCP2-null mice at full symptomatic stages, no changes in expression were observed at pre- or early symptomatic stages of development. This result further supports the hypothesis that mitochondrial expression or functional changes are progressive over time in MeCP2-deficient systems.

## Evidence for altered mitochondrial function in MeCP2-deficient cells

The differential expression of genes coding for subunits of the ETC complexes in RTT patients and MeCP2-deficient mouse models strongly suggest that the function of these complexes will be correspondingly altered in MeCP2-deficient systems. One likely alteration would be mitochondrial membrane potential. Mitochondrial membrane potential is created when protons are pumped from the mitochondrial matrix into the inter-membrane space as electrons pass through the ETC (Mitchell, 1961; Jastroch et al., 2010). An increased mitochondrial membrane potential is indicative of an increased rate of electron transport through the ETC, while a decreased potential is indicative of decreased ETC transport and could also be indicative of increased proton leak. In different cells from MeCP2-deficient mice, decreased mitochondrial membrane potential has been observed (Großer et al., 2012; Janc and Müller, 2014), suggestive of decreased ETC activity and/or increased proton leak. Functional studies have supported this, as Gibson et al. (2010) showed decreased cytochrome c oxidase activity (Complex IV) in SH-SY5Y cells following MeCP2 knockdown, and Gold et al. (2014) reported a marked decrease in the activities of Complexes II, Complex III and Complex IV in both cerebellum and skeletal muscle of symptomatic MeCP2-null mice. Additional studies have reported increased respiration rates—but lower mitochondrial efficiencies—in mitochondria isolated from acute MeCP2-null mouse hippocampal slices (Großer et al., 2012), along with decreased ATP levels in both MeCP2-null mice brains (Saywell et al., 2006), and in MeCP2-null microglia cells (Jin et al., 2015). Similarly, Kriaucionis et al. (2006) reported that symptomatic MeCP2-null mice display increased expression of ubiquinol-cytochrome c reductase core protein 1 (Uqcrc1), and increased uncouple respiration rates upstream of complex IV. From data obtained by Uqcrc1 over-expression in N2A cells, they speculated that such Uqcrc1 upregulation could exacerbate normal electron transfer rates in the complex III assembly, and underlie the increased uncoupled respiration rates they observed in the MeCP2-null brain samples (Kriaucionis et al., 2006). Taken together, these studies provide strong evidence that MeCP2 deficiency does compromise the ability of mitochondrial to carry out oxidative phosphorylation.

## How might MeCP2 dysfunction lead to impaired mitochondrial function?

The studies discussed above indicate clearly that the absence of MeCP2 affects mitochondrial function. Precisely how this arises remains unclear, but the available evidence does suggest a progressive cascade (Müller and Can, 2014). In MeCP2-deficient cells there appears to be an early dysregulation of the normal expression patterns for key nuclear and mitochondrial DNA-encoded factors that are essential for proper mitochondrial function. These changes (and likely others of currently unknown etiology) initially lead to mitochondrial hyperpolarization (Galloway and Yoon, 2012), which could be viewed as a compensatory attempt to increase ATP production in a system with diminished ATP synthesis efficiency and/or increased ATP demands. However, this hyperpolarization, in conjunction with impairments in specific ETC complexes, facilitates an overproduction of ROS that over time reinforces a vicious cycle of that eventually compromises mitochondrial function, and causes mitochondrial depolarization (Großer et al., 2012; Janc and Müller, 2014). When this state is reached, the ability of mitochondria to produce ATP is further diminished, and the capacity of ATP-dependent cellular mechanisms is compromised. The swelling of MeCP2-deficient mitochondria observed in different systems could reflect a byproduct to the increased demand on the system to provide sufficient ATP to maintain cellular homeostasis.

## A model for how ANT1 dysregulation could play a role in mitochondrial dysfunction

While the precise mechanisms that initiate this feed-forward process remain speculative, and could involve multiple entry points, there are intriguing data to suggest the adenine nucleotide transporter (ANT1) may be a potential mediator. ANT1 is a transmembrane protein responsible for the normal shuttling of ATP and ADP across the inner mitochondrial membrane (Forlani et al., 2010). Under normal conditions, MeCP2 binds to methyl CpG sites in the promoter region of the ANT1 gene, and together with the zinc finger epigenetic regulator yin-yang 1 (YY1), negatively regulates ANT1 expression. In the absence of MeCP2, ANT1 would become de-repressed, leading to an elevation in its expression. In fact, in MeCP2-null mouse brain, fibroblasts derived from RTT patients, and several MeCP2-null cell lines show a significant increase in ANT1 expression (Forlani et al., 2010). This is intriguing, as ANT1 represents a site at which proton leak back across the inner mitochondrial membrane can occur. In fact, up to two-thirds of the normal basal proton leak observed in normally functioning mitochondria occurs through ANT1 (Brand et al., 2005). Importantly, this leak is directly dependent upon ANT1 prevalence, and is not dependent of ANT1 catalytic activity (Jastroch et al., 2010). Since the magnitude of the proton leak correlates specifically with the abundance of ANT1 (Jastroch et al., 2010), the increase of ANT1 in MeCP2-null cells could represent an initiation site for an enhanced basal proton leak. Any such increase in proton leak via ANT1 would likely initiate a compensatory increase in ETC activity to maintain ATP production, as reported by Kriaucionis et al. (2006). However, such an increase in ETC activity would also lead to an increase the number of electrons liberated from Complexes I and III, and thus contribute to increased cellular ROS levels (Mailloux and Harper, 2012). The net result would be mitochondria with increased proton leak, accelerated electron transfer rates, increased electron leak, and decreased respiratory control ratios (Kriaucionis et al., 2006). Initially, mitochondria compensate to preserve functional efficiency, but over time, the cumulative effects overcome compensatory function and the mitochondria depolarize and swell. Such a cycle would predict that the MeCP2-deficient system has an intrinsically limited ability to respond to energy demands (e.g., the presence of higher than normal ADP levels), which restricts its capacity to properly increase ATP synthesis in specific times of need (Brand and Nicholls, 2011).

## Targeting mitochondrial dysfunction in MeCP2-null neurons

It is important to note that many RTT patients live into their 5th or 6th decade, and *MECP2* mutations are not generally associated with neurodegeneration (Tarquinio et al., 2015). These observations argue that MeCP2-deficient cells can meet most of their essential needs, but are unable to function within a normal homeostatic window, and display a “failure to thrive” phenotype rather than one progressing toward a degenerative endpoint. This therefore indicates that the degree of mitochondrial impairment in MeCP2-deficient cells is not as robust as seen in many mitochondrial diseases, which may well afford a greater chance for therapeutic success. In fact, the pattern of differential expression of both mitochondrial and nuclear genes encoding ETC complex components, and other salient mitochondrial factors in MeCP2-deficient systems, are consistent with the idea that stressed mitochondria are attempting to improve their functional efficiency to meet homeostatic needs. The fact that mitochondrial impairments are not pronounced in pre-symptomatic or early symptomatic MeCP2-deficient mice, but rather develop more concomitantly with symptomatic manifestations, suggest the observed mitochondrial impairments may be linked to overall disease progression. The model presented here is one of a “feed-forward” cycle that becomes active over time as oxidative stress levels accumulate. That is, loss of MeCP2 function causes deregulation of nuclear genes encoding key proteins required to maintain mitochondrial function within a normal range, and as a consequence, the mitochondria work harder but with diminished efficiency to meet energy demands. Over time, the elevated electron leak progressively increases cellular oxidative stress, which further impairs the functional capacity of mitochondria. Thus, therapies that globally target various aspects of mitochondrial function or mitochondrial ROS production and biogenesis could potentially delay symptomatic onset and/or severity if administered at early pre-symptomatic stages, and/or interrupt the cycle and improve existing symptoms if administered at later stages.

There are data that support this possibility. For example, the administration of Trolox, a vitamin E derivative, to MeCP2-null hippocampal slices decreased levels of oxidative burden, and improved both short- and long-term synaptic potentiation deficits of the local circuitry (Großer et al., 2012; Janc and Müller, 2014). In addition, Janc et al. (2016) recently reported that while the administration of Trolox to MeCP2-null mice did not improve their ambulatory or respiratory phenotypes, it did significantly reduce their levels of oxidative stress and improve their blood glucose levels, short-term synaptic plasticity, and exploratory behavior phenotypes. Further, the administration of ω-polyunsaturated fatty acids (ω-PUFAs), which provide anti-oxidative protection, significantly decreased the levels of oxidative stress markers in the blood of RTT patients, and reduced the severity of motor-related impairments, non-verbal communication deficits, and breathing abnormalities in the same patients (De Felice et al., 2012). The findings that interventions targeting excessive ROS in MeCP2-deficient systems and in RTT patients have provided tangible benefits supports the hypothesis that mitochondrial impairments linked to oxidative stress directly contribute to RTT pathogenesis, and are not simply secondary consequences that appear after full symptomatic progression has occurred (Müller and Can, 2014). These data therefore support exploring additional prospective therapies in RTT models, and potentially RTT patients, that are designed to attenuate oxidative stress, and/or improve the functional efficiency of mitochondria.

## Summary

Considerable data show impaired mitochondrial function occurs in MeCP2-deficient cells, and mechanisms through which these functional alterations arise beginning to be identified. Studies to date indicate that the loss of MeCP2 function negatively affects the signaling efficiency of a host of intracellular signaling pathways, the communicative ability of neuronal populations, the excitability of neural networks, and the functional efficiency of mitochondria. Each of these alterations could conceivably represent targets for therapeutic development toward treating RTT. The fact that anti-oxidant strategies has improved local neural circuit activity *in vitro* and perhaps *in vivo* in RTT patients suggests mitochondrial regulation represents a target that allows at least some functional rescue to be attained. Intriguingly, a role for Foxg1 in mitochondrial function has now been identified (Pancrazi et al., 2015), and heightened levels of oxidative stress markers have been reported in RTT patients with CDKL5 mutations (Pecorelli et al., 2011). While it remains unclear if there is mechanistic commonality between mitochondrial deficiencies in these conditions and those arising from MeCP2 absence, these data do suggest that impaired mitochondrial function may also play a role in atypical RTT cases. While additional work is needed to resolve the magnitude to which impaired mitochondrial function contributes to RTT pathogenesis, the recent data reviewed here strongly suggest a re-visitation of the “mitochondrial hypothesis of RTT,” and the prospects for interventions targeting mitochondria, is warranted.

## Author contributions

NS: Conducted literature review for manuscript, wrote draft of manuscript, prepared Tables, collated citations. AA: Edited manuscript, refined model hypothesis, and provided field expertise to project. LM: Assisted in model development, edited manuscript, and provided field expertise to project. JE: Supervised project, wrote final draft of manuscript, and provided field expertise to project.

## Funding

This work was supported by an operating grant from the Canadian Institutes of Health Research to JE [MOP-81104], and by a Mary Gertrude I'Anson Graduate Scholarship received by NS from the University of Toronto.

### Conflict of interest statement

The authors declare that the research was conducted in the absence of any commercial or financial relationships that could be construed as a potential conflict of interest.
